# Integrated economic and experimental framework for screening of primary recovery technologies for high cell density CHO cultures

**DOI:** 10.1002/biot.201500336

**Published:** 2016-05-09

**Authors:** Daria Popova, Adam Stonier, David Pain, Nigel J. Titchener‐Hooker, Suzanne S. Farid

**Affiliations:** ^1^ Department of Biochemical EngineeringUniversity College LondonLondonUK; ^2^ Lonza Biologics plcSloughBerkshireUK

**Keywords:** Bioseparation, High cell density, Mammalian cell culture, Primary recovery, Tangential flow filtration

## Abstract

Increases in mammalian cell culture titres and densities have placed significant demands on primary recovery operation performance. This article presents a methodology which aims to screen rapidly and evaluate primary recovery technologies for their scope for technically feasible and cost‐effective operation in the context of high cell density mammalian cell cultures. It was applied to assess the performance of current (centrifugation and depth filtration options) and alternative (tangential flow filtration (TFF)) primary recovery strategies. Cell culture test materials (CCTM) were generated to simulate the most demanding cell culture conditions selected as a screening challenge for the technologies. The performance of these technology options was assessed using lab scale and ultra scale‐down (USD) mimics requiring 25–110mL volumes for centrifugation and depth filtration and TFF screening experiments respectively. A centrifugation and depth filtration combination as well as both of the alternative technologies met the performance selection criteria. A detailed process economics evaluation was carried out at three scales of manufacturing (2,000L, 10,000L, 20,000L), where alternative primary recovery options were shown to potentially provide a more cost‐effective primary recovery process in the future. This assessment process and the study results can aid technology selection to identify the most effective option for a specific scenario.

**Abbreviations**
CCTM: cell culture test material
COG_PR_: cost of goods for primary recovery operations
MADM: multi‐attribute decision‐making
RMU: relative monetary units

## 1 Introduction

Major improvements in mammalian cell culture methods have been achieved over the last 20 years. Today, high titer fed‐batch processes have been reported to achieve up to 13 g/L, a ten‐fold increase since the mid 90s 1, 2. Meanwhile peak cell densities achieved using monoclonal antibody (mAb) producing CHO cell lines have reached over 200 × 10^6^ cell/mL 3 with average cell densities used in existing fed‐batch cell culture processes of approximately 10 to 20 × 10^6^ cells/mL 4.

Primary recovery operations in mammalian cell culture processes have typically been designed to provide high levels of solids removal collectively aiming to remove solids >0.1–0.2 µm in diameter. Centrifugation combined with depth filtration stages have been described as the current workhorses of primary recovery 5, 6, 7 in large‐scale manufacturing, typically achieving 98–99% solids removal prior to sterile filtration stages. However as cell culture performance has improved dramatically, primary recovery and purification operations have been facing increasingly challenging feedstreams, and it is unclear whether current unit operations can continue to provide feasible processing options in the future. This dilemma provided the impetus for the studies reported in this paper.

Most mammalian cell culture processes used today employ centrifuges fitted with hermetically‐sealed feed zones, which reduce the levels of shear exposure. However, energy dissipation levels still reach 0.019 × 10^6^ Wkg^−1^, which in some cases results in significant cell breakage, and subsequently impacts depth filter area requirements 8. The key parameter causing variation in solids removal performance at a fixed cell density is the cell culture viability at harvest. Generally, efficient solids removal can be achieved at viabilities >50% 4. For cell culture material with lower viabilities centrifugation is often considered to be more challenging.

At the turn of the century, cell densities by mammalian cell culture required relatively low centrifugal discharge frequencies and the contribution to product loss was considered minimal, especially when compared to those experienced in microbial processes 9.

Subsequent increases in cell densities have resulted in the solids loads approaching >10% v/v. Centrifuge efficiency at such high solids contents is generally lower and there is a need to desludge the centrifuge more frequently, potentially leading to a greater degree of product loss than previously witnessed.

Depth filtration has been used primarily for solids removal of supernatant post centrifugation, and in some cases (generally at scales <2000 L) to process material direct from cell culture. Most available depth filters use charged filtration media and have been demonstrated to achieve a level of DNA and host cell protein (HCP) removal 10, 11. Depth filters tend to be followed by absolute pore size rated filters (typically 0.45, 0.2 or 0.1 µm) which ensure the removal of solid particulates as well as some endotoxins and a degree of viral removal from the cell culture harvest material 12. Together these steps ensure a particle‐free product solution, which can then proceed successfully to packed bed chromatography steps.

A wide range of alternative technology options for primary recovery have been previously identified including flocculation 13, 14, 15, 16, 17, acid precipitation 18, 19, expanded bed absorption 20, 21, 22, counter current tangential chromatography 23 and alternating tangential flow filtration 24. Although these operations are expected to bring benefits in the future, current limitations in terms of practical application were reported for example potential issues with presence of flocculant in the bulk drug substance, low product yields and highly sensitive operational performance 6, 16. Tangential flow microfiltration (TFF) options, on the other hand, have been suggested in the past to deliver high processing rates without adverse effects on cell viability 25. This advantage can potentially play a key role in reducing cell damage during primary recovery of high cell density cell culture feeds, subsequently reducing potential impurity releases. Hollow fiber membranes have primarily been used in mammalian cell perfusion cultures, but are typically not considered for batch type harvest operations. The membrane costs of TFF can be lower than the costs of typical depth filtration media, especially where single use modules are required, as the TFF membrane media tends to be reusable 26.

The pressures to accommodate higher cell density feed streams during primary recovery provided the motivation for this paper in which the ability of both current and alternative primary recovery technologies to cope with predicted future feed profiles were evaluated. Cell culture test materials (CCTM) were used for this purpose. The method for CCTM generation allowed independent control of impurity, product, cell density and viability variables to create the perceived most demanding feed material conditions 27. The technologies were evaluated based on the following performance criteria: solids removal, yield and impurity removal. The technologies were then ranked using a multi‐attribute decision‐making (MADM) technique and the successful candidates were assessed further using an economic evaluation and facility fit criteria.

## 2 Materials and methods

### 2.1 Cell culture and cell harvest

Cell culture was carried out using the CY01 cell line, kindly donated by Lonza Biologics, in 5 L STRs (B. Braun BIOSTAT B‐DCU control unit, Sartorius, Epsom, UK) and harvested on day 13. Cell culture set points have been carried out as previously described in Popova et al. 27. Total cell concentrations at harvest were ∽9 × 10^6^ cells/mL with an average viability of 77%. The harvested material was used to generate cell culture test materials with the representative most challenging target conditions for future primary recovery feeds. These included as a cell density of 100 × 10^6^ cell/mL with a cell viability of 40%, IgG_1_ concentration of 20 g/L and a HCP concentration of 20 g/L. These conditions were selected using a survey compiling expert opinion on the likely future cell culture profiles to primary recovery. Additionally, a low viability was selected to provide a challenging case for the selected technologies. The CCTM generation was described previously in detail 27. The cell culture harvest was concentrated and spiked with the volumes of IgG_1_ and a HCP stock to create the required conditions. Apoptosis induced cell stock was added to the CCTM in order to achieve the target viability of 40%. All cell density and viability measurements were carried out using a ViCell™ (by trypan blue exclusion).

### 2.2 Primary recovery methods

Technologies were selected to represent current and alternative primary recovery options. Three centrifugation (hermetically‐sealed disc‐stack centrifuge) and primary depth filtration options (05SP, 10SP and 30ZA media options) were selected to represent the current options, where a moderate inlet flowrate to the centrifuge of 100 L/h was tested. The 05SP depth filtration medium typically removed particles of 2–10 µm, the 10SP medium removed particles between 1 and 5 µm and the 30ZA medium was positively charged and removed particles of 1–2 µm. Two tangential flow filtration options (0.45 μm microfilter Bio‐Optimal MF‐SL™ and a 0.22 μm anion exchange membrane QyuSpeedD™) were selected to represent the alternative options. The CCTM were then used as feed to these unit operations.

#### 2.2.1 USD centrifugation

Detailed methodologies for USD centrifugation have been described previously 8, 28, 29. A microwell plate‐based method described by Tait et al. 30 was used in this paper. A rotating shear device was used to mimic the shear experienced during the centrifugation step. Hutchinson et al. 28 previously correlated the high and low energy dissipation rates equivalent to non‐hermetic and hermetically‐sealed disc‐stack centrifuge feed zones as 0.37 × 10^6^ Wkg^−1^ and 0.019 × 10^6^ Wkg^−1^. As hermetically‐sealed feed zones are most commonly utilized for mammalian cell culture processing at present, only low shear centrifugation conditions were mimicked.

Sheared material was subsequently centrifuged using an Eppendorf 5810R bench top centrifuge (Cambridge, UK) with an A‐4‐62 swingout rotor. The fill volumes across the plate used were set to give an equivalent feed flow rate of 100 L/h in the centrifuge to represent a moderate flow rate into a medium scale centrifuge – the CSA‐1 (Westfalia, Oelde, Germany) with a sigma (Σ) value of 680 m^2^.

#### 2.2.2 USD depth filtration

Depth filtration media was kindly provided by 3M (Bracknell, UK) and was cut in‐house to provide a total effective area of 0.28 cm^2^ and inserted into a custom made manifold. Pressure was applied at 100 mbar using a vacuum manifold (Tecan VacS, Tecan, UK). Simultaneously a liquid handling arm (Freedom EVO^®^ liquid handling system, Tecan, UK) was set up to monitor and record the retentate volume throughout the filtration procedure, until the flux declined to 80% of the initial value. The scale comparison of this method has been discussed previously 31, 32.

The results were analyzed based on the *V*_max_ methodology assuming a gradual pore constriction model:

(1) $$\frac{t}{V}=\frac{\text{1}}{Q_{0}}+\left(\frac{\text{1}}{V_{\max}}\right)t$$

where *V* is the total filtrate volume collected over time *t*, *Q*_0_ is the initial flow rate, and *V*_max_ is the maximum volume that can be filtered before the filter is completely blocked and the flux reaches zero.

#### 2.2.3 USD tangential flow filtration

Bio‐Optimal MF‐SL™ and QyuSpeed D™ (QSD) (Asahi Kasei, Japan) hollow fiber modules with areas of 0.0004 m^2^ and 0.0006 m^2^ provided by a single fiber (15 cm height) were run at a constant flux of 30 LMH using an AKTA Crossflow device (GE Healthcare, Little Chalfont, UK). The manifold containing the single hollowfibre was custom made and kindly provided by Asahi Kasei. The initial feed flow rate was set to achieve a constant shear rate of 2300 s^−1^ for both module types, and the backpressure was maintained positive by using a manually operated valve when required. The module was wetted for 30 min prior to the start of the filtration using purified water.

### 2.3 Analytical techniques

#### 2.3.1 Solids removal performance quantification

The percentage solids removal post filtration operations was calculated based on optical density (OD) at 600 nm measurements of the feed solution (*F*_OD_), OD of the clarified sample (*S*_OD_) and normalized to a maximum primary recovery performance achieved by passing a clarified sample from each technology through a 0.2 µm PES syringe filter (*S*_100%_).

(2) $$S=\frac{F_{\mathrm{OD}}-S_{\mathrm{OD}}}{F_{\mathrm{OD}}-S_{\text{100}\%}}$$

OD measurements of the feed material were diluted with PBS and carried out at cell concentrations of 2 to 5 × 10^6^ cells/mL within the linear range of the instrument. OD measurements of the clarified material were not diluted.

Dry solids weight measurements were carried out by placing pre‐weighed snap‐top tubes with 2 mL of sample material at 80°C for 48 h, while allowing evaporation. Tube weight of the dehydrated material was recorded using a scale with a reliability of 0.0001 g (Sartorius Stedim, Epsom, UK).

#### 2.3.2 Impurity removal and concentration yield quantification

DNA and HCP were quantified in stock solutions as well as pre and post primary recovery operations. DNA concentration was measured using a Quant‐iT™ PicoGreen^®^ dsDNA Reagent Kit (Invitrogen, Paisley, UK). The BCA assay (ThermoFisher Scientific, Loughborough, UK) was used for quantifying relative HCP removal. Bovine Serum Albumin (ThermoFisher Scientific, Loughborough, UK) was used as a standard. HCP concentrations were calculated by subtracting the quantified IgG_1_ concentration value from the total protein value obtained using the BCA assay:

(3) $$\mathrm{HCP}=\mathrm{TP}-TY_{s}$$

where *TP* is the total protein concentration and *TY*_s_ is the total product concentration in the sample. IgG_1_ concentration was determined using a Protein G column (HiTrap™, GE Healthcare, UK) on an HPLC system (Agilent Technologies, UK), as previously described in Popova et al. 27. Concentration yield (*Y*_CP_) was calculated for the assessment of membrane performance at small scale using the following equation:

(4) $$Y_{\mathrm{CP}}=\frac{TY_{P}}{TY_{F}}$$

Where *TY*_P_ is the product concentration in the post processed material and *TY*_F_ is the product concentration in the feed material.

#### 2.3.3 Particle size distribution measurement

Particle size distribution of the clarified material has been carried out as previously described in Popova et al. 27.

#### 2.3.4 2D PAGE

Cell culture feed and samples post primary recovery were prepared using a 2D Clean‐Up Kit (GE Healthcare). 200 µg of total protein was loaded onto 7 cm IPGPhor strip (pH 3–10 Non‐Linear, GE Healthcare, UK). The second dimension was run using pre‐cast Bis‐Tris gels (4–20%, 7.0 × 7.0 × 0.1 cm ZOOM IPG Well). Staining was carried out using SyproRuby™ stain as per manufacturer's instructions. The images were scanned using a Typhoon 9400 laser scanner (GE Healthcare, UK). SameSpots software (TotalLab, Newcastle, UK) was used for image analysis. Normalized spot volumes were calculated and compared across the gels.

### 2.4 Performance attribute ranking and assessment methodology

A weighted sum multi‐attribute decision making (MADM) technique 33, 34, 35, 36 was used to combine the performance data into a single metric. Initially, the calculated technology performance values for each attribute were normalized to a 0–1 scale, where the zero value represented the worst case performance result and value of one represented the best case performance result.

(5) $$N=\frac{P_{A}-P_{\min}}{P_{\max}-P_{\min}}$$

where *P*_A_ is the actual figure for a performance attribute (e.g. solids removal), *P*_min_ is the minimum value achieved by the technologies and *P*_max_ is the maximum value achieved by the technologies for the same performance attribute. The normalized values were then weighted using a ratio of 3 : 2 : 1 for the performance attributes of solids removal to DNA removal to HCP removal. This ratio was selected based on a survey carried out to quantify industry opinion on demands facing primary recovery operations in the future (data not included). The subsequent sum of the weighted values for each technology leads to an overall normalized rank figure (*OR*_N_) from which technology performances can be compared.

(6) $$OR_{i}=\sum \omega_{i}N_{i}$$

(7) $$OR_{N}=\frac{OR_{i}-OR_{\min}}{OR_{\max}-OR_{\min}}$$

where ω_i_ is the weighted value and the *N*_i_ is the normalized value each corresponding to the performance of metric *i*. The resultant *OR*_N_ also has a value between zero and one, therefore representing the least and the most efficient option for a given scenario respectively. Selection criteria of the current typical minimum performance in terms of solids removal, yield and impurity removal were used as cut off criteria for technology performance.

### 2.5 Economic evaluation methodology

The economic evaluation was focused on the primary recovery stages only using the same worst case input conditions as were generated in the practical experiments (Supporting information, Table S1). These were combined with the sizing data collected during the experimentation and further assumptions (Supporting information, Tables S2 and S3) to calculate equipment duties required, kg product outputs per batch, capital investment and cost of goods (COG_PR_) outputs for the primary recovery operations for three scale scenarios: 2000 L, 10 000 L and 20 000 L production scales. A detailed process economics model was built in Excel (version 2010) that integrated mass balance, design and cost equations so as to generate the key performance metrics for the different primary recovery strategies. The mass balance and design equations accounted for features such as the impact of the cell density on the number of centrifugation discharges required (calculated based on the centrifuge model used to obtain experimental data) and the resulting yield loss. Experimental results were used as inputs for worst case filter throughputs and flux likely to be achieved. The cost equations were similar to those detailed in Farid et al. 37.

#### 2.5.1 Economic evaluation assumptions

Primary recovery operations were defined as those activities involved in the processing of cell culture material during cell culture harvest until the completion of the sterile filtration stage prior to chromatographic purification. Sizing data including throughput, yield indicator and solids removal were collected using the experimental set up described in section 2.2. This was combined with additional scenario constraints to provide the final sizing outputs. The scenario constraints included a target processing time of 6 h. In addition, details on equipment performance at the selected scale were constricted to specific equipment choices. This included a choice of Alpha Laval BTAX215H and Alpha Laval BTAX205 centrifuges, lenticular mobile skids for depth filtration, 8 and 5 m^2^ modules for the Bio‐Optimal MF SL™ and the QSD™ options respectively.

Production was assumed to consist of 17 batches per year, with a process length assumed to be 20 days. The COG_PR_ comprised of both the direct and indirect costs for primary recovery operations only, assuming use of an existing facility. The direct costs included materials (e.g. filters, single use materials etc.), labor costs (including operational labor) and WFI costs. Labor costs were derived by assuming a maximum shift length of 8 h as well as a requirement of one operator per large scale rig. The indirect costs included depreciation of 10% per annum over 10 years based on the capital investment. Capital investment was calculated on reusable equipment (e.g. filtration skids, centrifuge units etc.) and auxiliary equipment (e.g. pumps) using Lang factors shown in Supporting information, Table S4. In order to capture the necessary changes involved in the installation of new equipment at a manufacutirng site, a Lang factor value of 1 was assigned to current technology options. Lang factors capturing the costs involved in the installation of alternative equipment were estimated to total a value of 3.45.

## 3 Results and discussion

### 3.1 Yield, particle size distribution and impurity removal comparison

**Figure 1:**
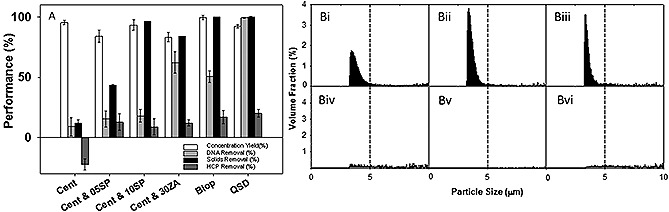
(**A**) Primary recovery technology performance under the worst case screening study conditions generated using the CCTM in terms of % concentration yield, % DNA removal performance, % solids removal and % HCP removal. Vertical error bars show the standard deviation of technical replicates (*n* = 3). (**B**) Particle size distributions of samples post solids removal using the different primary recovery technology options (**B i**) supernatant fraction post USD centrifugation, (**B ii**) permeate fraction post centrifugation followed by depth filtration using 05SP media, (**B iii**) permeate fraction post centrifugation followed by depth filtration using 10SP media, (**B iv**) permeate fraction post centrifugation followed by depth filtration using 30ZA media, (B v) permeate fraction post tangential flow filtration using Bio‐Optimal MF‐SL™, (**B vi**) permeate fraction using QSD™ in tangential flow filtration mode. The average distributions are plotted from *n* = 5 technical replicates.

The performance of three centrifugation and depth filtration options (05SP, 10SP and 30ZA media) as well as two tangential flow filtration unit options (Bio‐Optimal MF‐SL™ and QSD™) were investigated when challenged with the most demanding cell culture material conditions (Fig. 1A). A USD set up was used to mimic the characteristics of a disk‐stack centrifuge operating with a hermetically‐sealed feed zone and a feed flow rate of 100 L/h. The centrifuged material was then passed onto the USD depth filtration set up using a 0.28 cm^2 ^depth filter disc of each of the three media types at a constant pressure of 100 mbar. The resultant filtrate was compared to the material which passed through the single fiber set up of the Bio‐Optimal MF‐SL™ module (4 cm^2^) and the QSD™ (6 cm^2^) at 30 LMH constant flux. Comparisons were made in terms of solids removal, concentration yield and levels of impurity removal achieved.

Concentration yield across the technology options was found to be >80%. The observed product loss may have been due to non‐specific binding to the cellular and debris material as well as the tested membrane surface. No significant product loss was seen over time in unprocessed material used as control, at time scales of <5 h (data not shown). Initially solids removal was measured using OD measurements and subsequent calculation of the % solids removal, but this proved unreliable due to the high levels of fine particles generated during the USD centrifugation step. Therefore solids removal for the USD centrifugation step was further analyzed by dry cell weight measurement of the supernatant compared to the feed and was also normalized using the supernatant passed through a 0.22 µm filter. This presented a more reliable measure of the centrifugation performance. Solids removal was measured for the depth filtration options using the OD measurement methodology and was used as a rough filter performance indicator. Direct comparison between centrifugation and depth filtration solids removal capacities was not made due to the differences in the quantification techniques applied.

A typical particle size distribution profile of the CY01 cell line has been previously shown, where cell debris were found in the range of 2–5 μm, cells (including apoptotic and live) >5 μm 27, 30. HCP release into the supernatant stream was also observed, indicating high levels of cell damage due to shear. The particle size distribution data (Fig. 1B) of the feed material and the supernatant also showed an increase in particles between 2–5 µm in diameter, and the presence of some particles between 5–10 µm in diameter. An absence of particles with diameters above 10 µm in the supernatant stream, was observed. The 30ZA option however provided the highest degree of solids removal compared to the 5SP and 10SP options, as it has the smallest nominal pore size. However, breakthrough occurred during filtration and this increased the sample pool OD. A ∽10% total HCP removal was observed when using all three media types, as well as substantial DNA removal of 15–20% and 63% using the SP and the ZA media respectively. The observed difference in removal was expected and can be explained by the positive charge provided by the ZA media compared to the SP.

The Bio‐Optimal MF‐SL™ and the QSD™ options achieved >99% solids removal, however their performance differed in terms of impurity removal levels. Operating with the Bio‐Optimal MF‐SL™ provided a ∽50% DNA removal compared to >99% DNA removal using the QSD™ and HCP removal of 15 and 20% respectively. The Bio‐Optimal MF‐SL™ media is however uncharged and therefore was not expected to result in a high level of DNA removal. However high cell mass within the hollow fiber may have resulted in entrapment of DNA due to a combination of physical particle retention and non‐specific binding to the cellular matter. The QSD™ was expected to achieve a high level of DNA removal due to the high charge capacity provided by the diethylamine (DEA, NH([CH_2_CH_3_]_2_) ligands grafted to the polyethylene (PE) membrane material 38.

In terms of particle size distribution, the Bio‐Optimal MF‐SL™ showed the lowest presence of solids across the particle size range. The QSD, Bio‐Optimal as well as the centrifugation and 30ZA options showed an overall low solids content, with the solids fraction remaining below 3%.

### 3.2 HCP removal profile comparison

**Figure 2:**
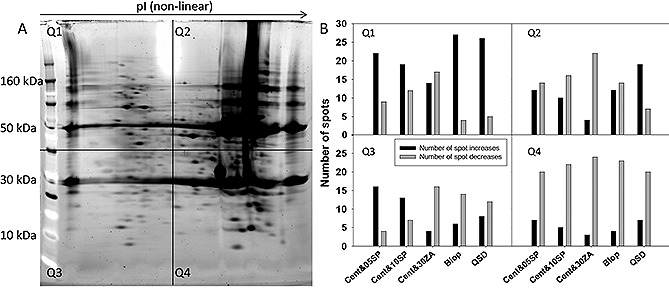
2D PAGE gel analysis of CCTM feed and samples post primary recovery using each of the selected technologies. The gels were divided into four quadrants based on gel coordinates. (**A**) 2D PAGE gel of the CCTM feed divided into four quadrants – (**Q1**) low pI and low molecular weight region, (**Q2**) high pI and low molecular weight region, (**Q3**) low pI and high molecular weight region, (**Q4**) high pI and high molecular weight region. (**B**) Increases and decreases in the normalized spot volumes compared to the CCTM feed in each quadrant using SameSpots software. Results were calculated using the average of three technical replicates for each of the tested conditions.

One of the key functions of the unit operations following the solids removal/clarification stages is HCP removal. Protein A typically removes ∽95–99% of HCPs 39. However, HCP reduction prior to protein A may have benefits in terms of protein A resin lifetime. In addition, specific HCP removal may still be a concern for some processes or cases where upstream batch‐to‐batch variability may cause expression of HCPs which are particularly difficult to remove. Therefore, the HCP removal potential of each technology option was investigated further in order to gain an understanding of the types of HCPs these technologies tend to remove. 2D PAGE gels of the starting material and post primary recovery operations were run in triplicate. HCP normalized spot volumes were compared to the CCTM gels in four gel areas numbered Q1‐Q4 (Fig. 2A), representing different pI and molecular weight combinations (low pI and low molecular weight; high pI and low molecular weight; low pI and high molecular weight; high pI and high molecular weight). Spot increases and decreases were calculated relative to the CCTM gels and included any new spots which were not originally found on the CCTM gel images (Fig. 2B). This method was used to indicate any specific areas of HCP removal which a particular technology can provide. Highest spot number decreases were seen in the low molecular weight and high pI region across all the technologies (Q4). This may be due to some association of these HCP positively charged HCPs to the cell debris accumulating on the retentate size of the tested membranes. Centrifugation plus 30ZA, Bio‐Optimal MF SL™ and QSD™ options also showed higher HCP removal in the Q3 region. Highest spot increases were observed in the Q1 region for all the technologies except the centrifugation plus 30ZA option. The centrifugation and 30ZA option showed higher net spot decreases than increases across all the quadrants.

A dark line of unresolved lower pI HCPs can be seen on the feed material gel (Fig. 2A). Due to the poor resolution these were not quantified, but a significant reduction of this region was observed post QSD™ application and some reduction was observed post Bio‐Optimal MF‐SL™ application (images not shown).

### 3.3 Performance attribute ranking and assessment

An MADM additive weighting technique was used to assess the practical findings and quantify the overall performance by considering the performance attributes taken together. A potential scenario for the selection criteria was then explored, where yield and purity targets were selected based on the current platform processes as well as literature data.

**Figure 3:**
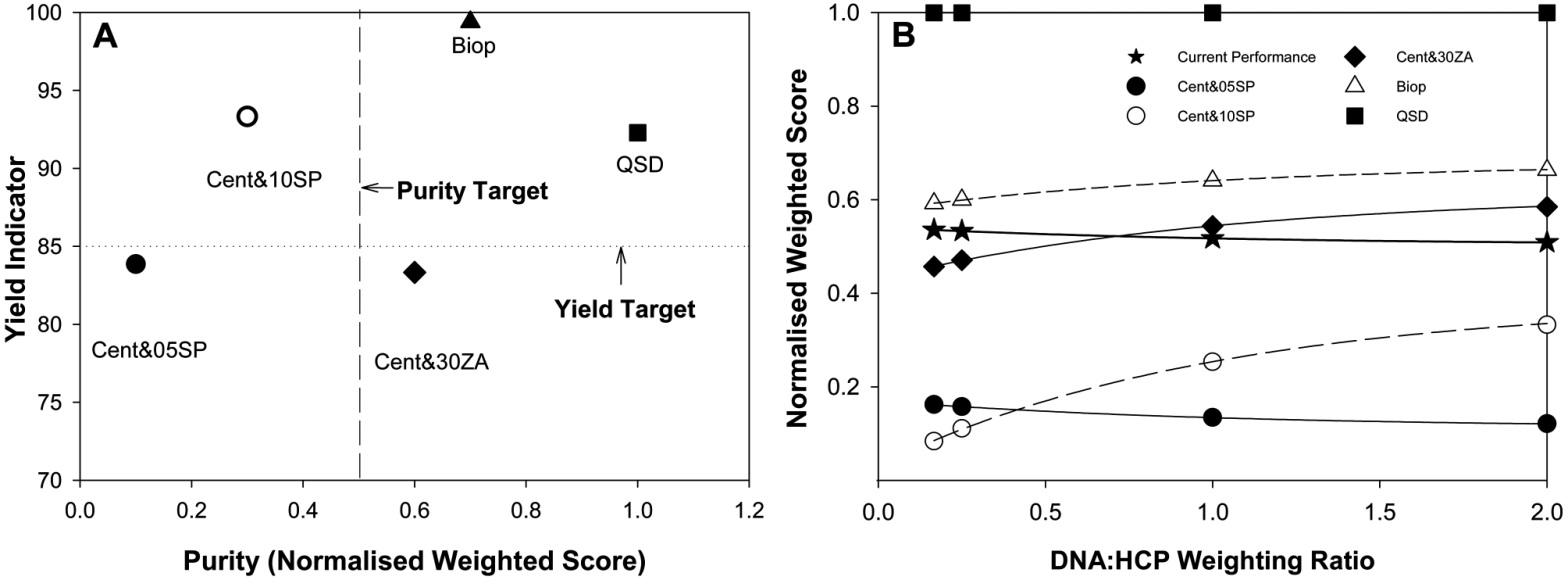
(**A**) Primary recovery technology performance scores calculated using an MADM additive weighting technique. The normalized weighted score was calculated for purity by assuming a 3 : 2 : 1 weighting ratio of solids removal : DNA removal : HCP removal. These scores are presented for centrifugation plus 05SP depth filtration media option, centrifugation plus 10SP depth filtration media option, centrifugation plus 30ZA depth filtration media option Bio‐Optimal‐MF‐SL™ option, and the QSD™ option. The scores were plotted against the yield result obtained in terms of product concentration. Technology performance targets were applied based on existing processes to obtain a yield target and a purity target for technology selection. (**B**) Sensitivity analysis on score weighting performed by altering the DNA : HCP weighting ratio. Normalized weighted score for the selection criteria rating based on current operational performance.

Solids removal, DNA reduction and HCP reduction results were normalized based on the minimum and maximum values for each attribute to obtain a rating value between zero and one (Supporting information, Table S1). The example values for a current platform are also shown. The ratings were then obtained for each technology and each of the selected performance attributes. An overall purity weighted score was then obtained and compared across the technologies (Supporting information, Table S5). These results reflected the practical data, where the QSD™ displayed the highest score, followed by Bio‐Optimal MF‐SL™, the centrifugation plus 30ZA, centrifugation plus 10SP and finally centrifugation plus 05SP option. Based on the normalized weighted score of the ”current operational level“ selected, centrifugation plus 05SP as well as centrifugation plus 10SP options were seen to fall below the desired level, while centrifugation plus 30ZA, Bio‐Optimal MF‐SL™ and the QSD™ options performed above the set base line. Additional minimum yield criteria were then also implemented and set to be 90% (Fig. 3A). As a result the Bio‐Optimal and the QSD™ technologies met both of the set criteria while the centrifugation plus 30ZA option met the target purity criteria but not the yield criteria. The centrifugation plus 10SP option met the yield criteria but not the purity criteria and the centrifugation plus 05SP option met neither of the criteria. If reducing the acceptable yield criteria to 80% is possible, the centrifugation plus 30ZA option would not be ruled out. Accounting for this possibility we did not rule out the centrifugation plus 30ZA option at this stage.

**Figure 4:**
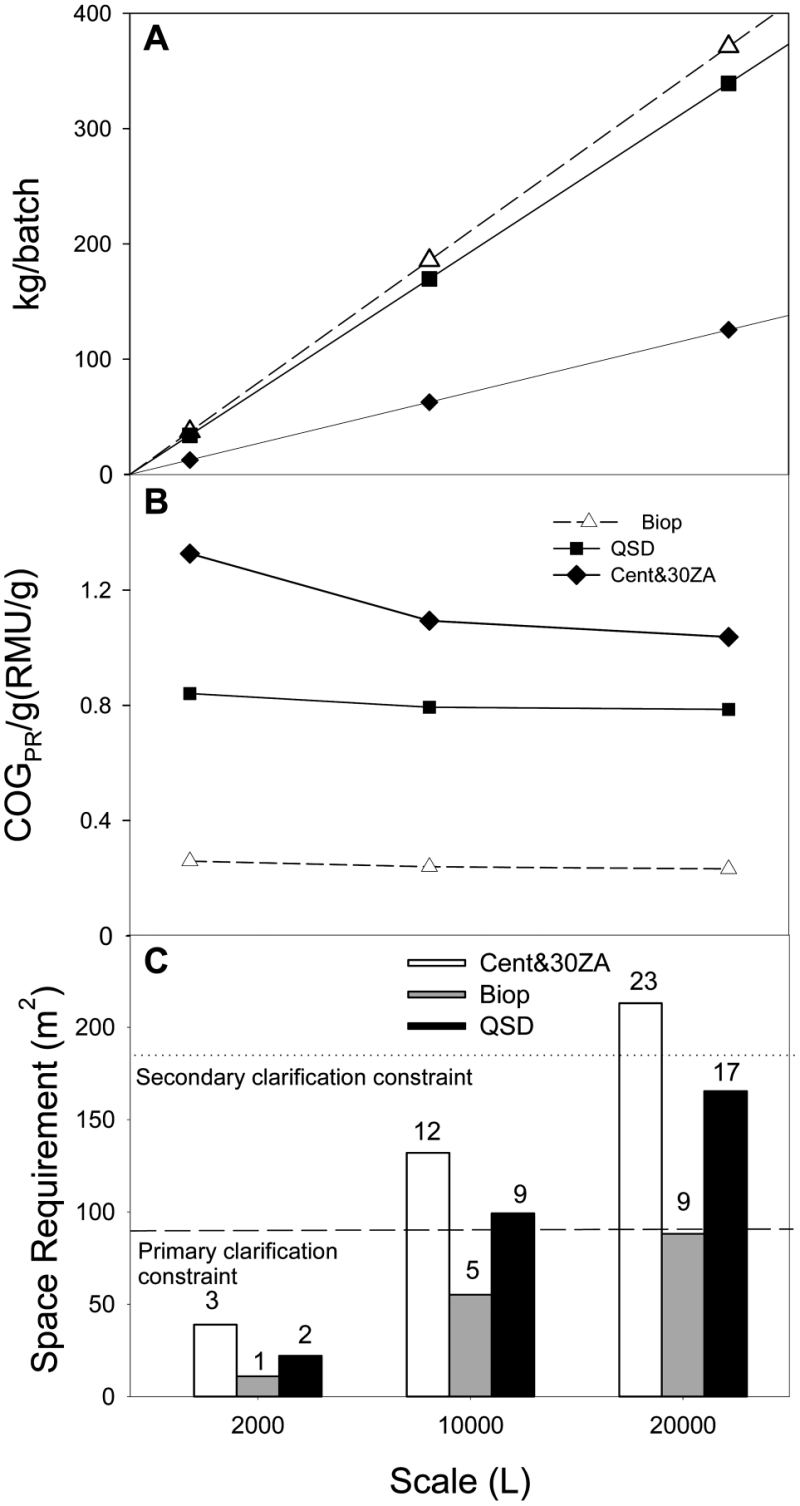
Kilogram per batch output (**A**), COG_PR_/g (**B**) and floor space required (**C**) for the primary recovery technology options at three scale scenarios of 2000 L, 10 000 L and 20 000 L. Analysis based on experimental performance at the selected worst case feed to primary recovery conditions, assuming 17 batches per year production at each scale. Cost of goods (COG_PR_/g) account for primary recovery costs only. Floor space considerations assumed a minimum of 1 m operational space around each unit. Results are presented for the centrifugation plus 30ZA option Bio‐Optimal MF‐SL™ option ; QSD™ option, centrifugation plus 30ZA (primary clarification only) Bio‐Optimal MF‐SL™ QSD™; In (**C**), the numbers above the bars indicate the number of filtration skids required in each given scenario. An example of an existing current worst case primary recovery space requirement for a 20 000 L process is indicated for primary clarification (– – –) and primary and secondary clarification collectively (········).

A sensitivity analysis was performed on the weighting ratio of DNA : HCP removal to determine the impact on the ranking of the technologies. Fig. 3B illustrates that if the HCP removal score is considered more important than DNA removal (DNA : HCP removal weighting ratio <0.75), the normalized weighted score of the centrifugation plus 30ZA option falls below the acceptable threshold values. The ranking of centrifugation plus 05SP and centrifugation plus 10SP options also switches at a DNA : HCP removal ratio below 0.25 where centrifugation plus 10SP option scores fall below the 05SP alternative. These trends are driven by the very small differences in HCP removal performances of the technologies and the much greater differences in DNA removal performances.

### 3.4 Cost of goods comparison

In order to compare the economic impact of the primary recovery technology selection, the cost of goods was calculated for the primary recovery operations defined as all activities post cell culture harvest, not including protein A purification operation and beyond.

Throughput data for the three depth filtration options and the Bio‐Optimal MF‐SL™ and the QSD™ was used in the comparison. The Bio‐Optimal MF‐SL™ was found to provide the highest capacity of >110 L/m^2^, followed by the 05SP, 10SP QSD™ and 30ZA media (101, 88, 82, 32 L/m^2^ respectively). This is consistent with a previously reported throughput range seen in the SP filtration media when processing high cell density material 40.

This data together with the unit operation assumptions and the performance data presented in the previous section provided the inputs to the cost of goods model (Supporting information, Table S2). Based on the technology performance results, economic outcomes are presented for those technology options which fulfilled one or more performance target criteria (i.e. centrifugation plus 30ZA; Bio‐Optimal MF‐SL™; QSD™). The centrifugation plus 30ZA media option achieved the lowest kg/batch output of product, when compared to both of the TFF options (Fig. 4A). This low output was due to the yield losses expected during the large scale centrifugation operation. At such high solids concentrations, the number of discharge operations required by the selected centrifuge models, reduced the overall step yield from ∽90% to 30–40% at the three scale scenarios. This had a considerable impact on the cost of goods (RMU/g) output (Fig. 4B), across the scales where the centrifugation plus 30ZA option is the most costly followed by the QSD™ and the Bio‐Optimal MF‐SL™. The RMU/g figures were benchmarked against a commercially available software (BioSolve, BioPharm Services, Bucks, UK), which yielded results within the same order of magnitude.

The space required to facilitate the use of the equipment required was also investigated (Fig. 4C). The centrifugation plus 30ZA media was found to require >200 m^2^ of floor space to accommodate the skids at the 20 000 L scale, which totalled 23 units. A current 20 000 L facility was used as a benchmark to provide facility fit constraints. It can accommodate 10 skids for primary clarification filters and 10 skids for secondary clarification filters (as the current worst case scenario). The space required to accommodate the 30ZA primary clarification skids at the 20 000 L scale at the set scenario conditions was more than double this. Although the number of skids required to accommodate the QSD^TM ^option was also high, secondary filtration prior to the sterile filters is not required, and would fit into this hypothetical facility. The Bio‐Optimal MF‐SL™ option required approximately a quarter of this floor space. This indicates that in cases where existing facilities are used for new processes or alternative unit operations, the TFF options may prove to be more easily accommodated.

**Figure 5:**
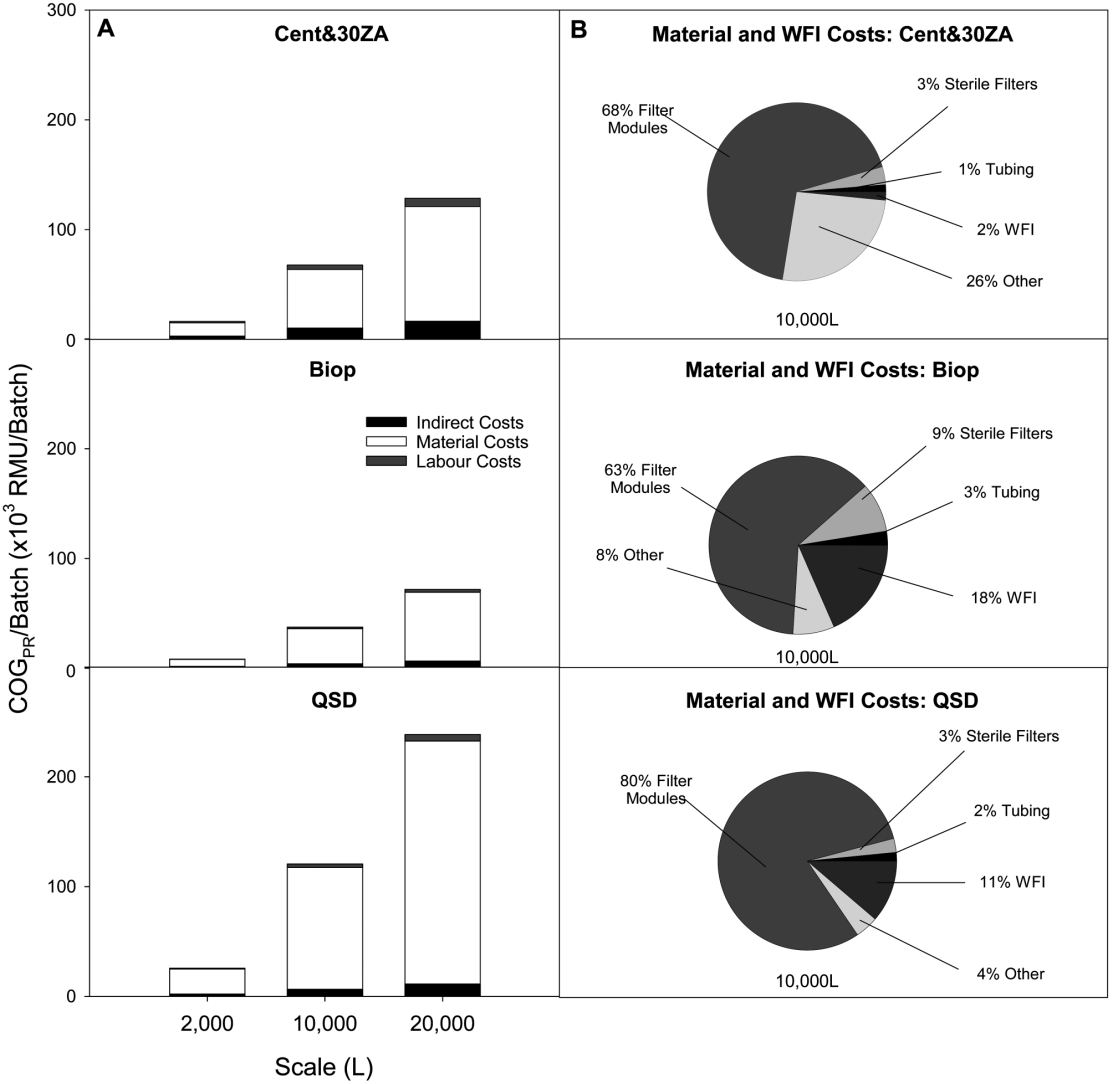
(**A**) Comparison of COG_PR_ breakdown at each scale scenario on a category basis for indirect costs , material costs and labor costs, for the primary recovery operations only. (**B**) Material and WFI costs breakdown at the10 000 L scale scenario.

Material costs dominate the COG_PR_/batch in all technology cases across the scales (Fig. 5A). The sizing of the depth filtration and TFF modules in each scenario were ensured to fit processing time criteria of process completion within 6 h, allowing for 2 h for CIP of the centrifuge and the TFF rigs. For the purpose of these calculations it was assumed that one operator will be required for each rig in the processing facility. The increased labor costs in the case of centrifugation plus 30ZA reflect the depth filtration rig requirement in comparison to the Bio‐Optimal MF‐SL™ and QSD™ options.

The QSD™ option had the highest cost per batch, which is approximately double the cost of the centrifugation plus 30ZA option and over three times the cost of Bio‐Optimal MF‐SL™. This is due to the high cost per module, as the technology is designed and priced to compete with anion exchange resins and membranes (Supporting information, Table S3). The QSD™ module cost comprises 80% of the total COG_PR_/batch compared to the 60–70% in the centrifugation plus 30ZA and Bio‐Optimal MF‐SLTM cases (Fig. 5B). In addition, WFI costs make up 10–20% of the batch cost in the case of the TFF options compared to 2% in the case of centrifugation plus 30ZA.

The lowest cost operation across the scale scenarios is the Bio‐Optimal MF‐SL™, due to the high throughput it achieves and the relatively low cost of the modules compared to the QSD™. The base case reusability assumed for both TFF options was 10 times. However increasing membrane reusability beyond 50 times significantly reduces the RMU/g output. However, options including centrifugation can benefit from economies of scale once a larger centrifuge model is required (scales >5000 L).

## 4 Concluding remarks

Using a combination of cell culture test materials (CCTM), ultra scale‐down technologies, multi‐attribute decision‐making methods, process economics and facility fit considerations, this paper has demonstrated a methodology and results for achieving a screening of current and alternative primary recovery technologies. The example technologies tested included three centrifugation and depth filtration options (using 05SP, 10SP and 30ZA media), and two alternative tangential flow filtration options (using Bio‐Optimal MF‐SL™ and QSD™ hollow fiber modules). Similar HCP removal levels were reached across all the tested technologies, however removal of specific HCP groups varied. Up to 99% DNA removal was achieved using the QSD™, with lower levels of DNA removal using the other options.

MADM analysis as well as selection based on current technology performance criteria showed that only two options met the yield and purity criteria: Bio‐Optimal MF‐SL™ and the QSD™. The centrifugation plus the 30ZA option met the purity criteria but not the yield criteria. The options were further evaluated based on their economic performance. This showed the centrifugation plus 30ZA option to be the least cost‐effective across the 2000 L, 10 000 L and 20 000 L scale scenarios and not fit the facility constraints set based on a typical existing large‐scale facility. The Bio‐Optimal MF‐SL™ option was the most cost‐effective option across the 2000–20 000 L scales of operation.

Economic and MADM analysis of the alternative technologies has been used to identify primary recovery options for the future. The QSD™ was found to provide greater capability for DNA removal prior to purification operations, however remained a costly alternative. The Bio‐Optimal MF‐SL™ offered a similar level of solids removal but was more cost‐effective. Specific process requirements as well as other technology alternatives have each to be taken into account to further select a cost‐effective and appropriate technology choice.

## Supporting information

As a service to our authors and readers, this journal provides supporting information supplied by the authors. Such materials are peer reviewed and may be re‐organized for online delivery, but are not copy‐edited or typeset. Technical support issues arising from supporting information (other than missing files) should be addressed to the authors.

Supporting Information
