# Supplementary material for: Integrated economic and experimental framework for screening of primary recovery technologies for high cell density CHO cultures
Source: Biotechnol J. 2016 May 9;11(7):899–909. doi: 10.1002/biot.201500336 (PMC4999028; doi:10.1002/biot.201500336)
Supplement: Supplementary file 1 — Supporting Information [file BIOT-11-899-s001.pdf]

Biotechnology
Journal

Supporting Information for DOI 10.1002/biot.201500336

Integrated economic and experimental framework for screening of primary recovery technologies for high cell density CHO cultures

Daria Popova, Adam Stonier, David Pain, Nigel J. Titchener-Hooker, Suzanne S. Farid

© 2016 Wiley-VCH Verlag GmbH & Co. KGaA, Weinheim

Supporting Information Table S1: *Values representing typical existing operational performance for primary recovery technologies. These were combined with the projected technology performance results to obtain normalised technology performance ratings using minimum and maximum values in respective performance categories.*

Performance attribute	Current operational performance	Min value (%)	Max value (%)
Solids removal (%)	98	43	100
DNA Removal (%)	30	15	62
HCP Removal (%)	10	9	17

Supporting Information Table S2: *Normalised performance ratings (0-1) for each primary recovery technology option.*

Primary recovery options	Solids removal rating	DNA removal rating	HCP Removal Rating	Overall Weighted Score	Normalised Weighted Score
Current Operation Level	1.0	0.0	0.1	3.1	0.5
Centrifugation plus 05SP	0.0	0.2	0.3	0.7	0.1
Centrifugation plus 10SP	0.0	1.0	0.0	2.0	0.3
Centrifugation plus 30ZA	0.6	0.8	0.3	3.5	0.6
Bio- Optimal MF-SL™	0.4	1.0	0.7	4.0	0.7
QSD™	1.0	1.0	1.0	6.0	1.0

Note: The normalised values were calculated, using minimum and maximum performance data obtained experimentally. Performance categories were weighted 3:2:1 (solids removal: DNA removal: HCP removal) in order to obtain a normalised weighted score for each technology.

Supporting Information Table S3: *Key assumptions and calculations used to construct the process economics model.*

**Unit Operation Inputs**	**Source**	**Outputs**	**Calculations**
**Centrifugation**			
Cell culture volume, V_T	Scenario input		
Wet mass per cell, M_{WC}	Experimentally determined	Cell mass in the feed	$TCD \times V_T \times M_{WC}$
% of solids fraction remaining, S_R	Experimentally determined using particle size distribution	Solids mass in the sediment S_T	$(100-S_R)/100$
Centrifuge models available:<br>- Alpha Laval BTPX-205<br>- Alpha Laval BTAX215H	Scenario input		
Solids holding capacity, S_H			
Dewatering level – 50%	Manufacturer's specification	Mass of liquor in the sediment, M_{LW}	$0.5(S_H)$
Number of centrifuge units, U	Facility specification, = 1 specified unit	Number of discharge operations, N_P	$(S_T/S_H)/N_U$
Maximum feed flow rate, Q_{Cin}	Manufacturer's specification	Total liquor loss in the sediment V_W	$((N= x V_W)-(S_T/\rho_S))$ x U
Product is only lost in the liquor fraction of the sediment	Assumption	Processing time, t_{PCent}	$V_T/(U x Q_{Cin})$
**Depth Filtration**			
Product mass in, m_{Pin}	Input from centrifugation calculations	Volume in, V_{DFin}	$V_T - V_W$
Throughput V_{max}	Experimentally determined		
Safety factor S_F	Assumption = 1.5	Total membrane area required, A_{DF}	$(V_{DFin}/V_{max}) \times S_F$
Membrane area per module, A_M	Manufacturer's specification	Number of modules required, N_m	A_{DF}/A_M
Number of modules per skid, N_{MSK}	Manufacturer's specification	Number of skids required, N_{SK}	N_m/N_{MSK}
Hold up volume per module V_{DFhup}	Manufacturer's specification	Permeate volume, V_{DFp}	$V_{DFin}-(V_{DFhup} \times N_m)$
Concentration yield, Y_{CP}	Experimentally determined	Product Yield, Y_{DF}	$m_{Pin} /(m_{Pin}/ V_{DFin} \times (Y_{CP}/100) \times V_{DFp})$
Skid size: length and width, l,w	Manufacturer's specification		
Minimum space required for operation, OP_A	Assumption = 1m	Floor space required, A_{FSP}	$((l+ OP_A)x(w+ OP_A)) \times N_{SK}$
**TFF**			
Cell culture volume, V_T	Scenario input		
Throughput, T	Experimentally determined		
Safety Factor, S_F	Assumption = 1.5	Total membrane area required, A_{TFF}	$(V_T/T) \times S_F$
Membrane area per module, A_M	Manufacturer's specification	Number of modules required, N_m	A_{TFF}/A_M
Number of modules per skid, N_{MSK}	Manufacturer's specification	Number of skids required, N_{SK}	N_m/N_{MSK}
Hold up volume per module V_{TFFhup}	Manufacturer's specification	Permeate volume, V_{TFFp}	$(A_{TFF}/A_M) \times V_{TFFhup}$
Concentration yield, Y_{CP}	Experimentally determined	Product Yield, Y_{TFF}	$m_{Pin} /(m_{Pin}/ V_{DFin} \times (Y_{CP}/100) \times V_{TFF})$
Skid size: length and width, l,w	Manufacturer's specification		
Minimum space required for operation, OP_A	Assumption = 1m	Floor space required A_{FSP}	$((l+ OP_A)x(w+ OP_A)) \times N_{SK}$

**Cost of Goods Model Breakdown**		
**Cost Category**		**Value**
Direct Cost	Materials:<br>Filter Modules<br>Sterile Filters<br>Tubing<br>Miscellaneous Materials<br>Labour<br>Operating Labour<br>WFI	*f(utilisation)*
Indirect Cost	Maintenance<br>Depreciation<br>General Utilities (HVAC)	*0.1xCapital investment x Project duration*<br>*Capital investment/Depreciation period x Project duration*<br>*Cost per unit area x Facility size x Project duration*

Supporting Information Table S4: *Key cost assumptions used in the construction of the process economic model.*

Resource	Specification	£/Unit	Unit
**Equipment**			
Stainless steel vessel	2,000 L	65,700	£/unit
	10,000 L	71,500	
	20,000 L	174,200	
Peristaltic pump	2,000 L/h	2,750	£/unit
Alpha Laval BTX-205 centrifuge	2,500L/h	150,000	£/unit
Alpha Laval BTAX 215H centrifuge	12,000L/h	577,420	£/unit
Depth Filtration Rig	Standalone Rig System	20,000	£/unit
TFF Rig	Mobile skid	30,000	£/unit
**Labour**			
Operating labour	Single operator with maximum shift length of 8h	300	£/h
**Materials**			
Depth Filtration 30ZA modules	3.2m^2 per module	400	£/module
TFF: Bio-Optimal MF-SL modules	8m^2 per module	9,847	£/module
TFF: QSD modules	5m^2 per module	20,520	
WFI	Generated in-house assumed	100	£/L
Tubing		10	£/m
Sterile filter	0.22 µm	200	£/m^2

Supporting Information Table S5: Bioprocess facility capital investment factors and corresponding Lang factor included in the indirect cost calculations.

Lang Factor Description	Cent&30ZA<br>Base Case	TFF<br>Reusable Technology
Total Equipment Purchase Cost (inc. utilities)	-	1
Pipework & installation	-	0.9
Process control	-	0.37
Instrumentation	-	0.6
Commissioning	-	0.07
Equipment Validation	-	0.06
Contingency factor	-	1.15
**Lang factor**	**1.00**	**3.45**
